# Supplementary material for: Secondary structures that regulate mRNA translation provide insights for ASO-mediated modulation of cardiac hypertrophy
Source: Nat Commun. 2023 Oct 3;14:6166. doi: 10.1038/s41467-023-41799-1 (PMC10547706; doi:10.1038/s41467-023-41799-1)
Supplement: Supplementary file 1 — Supplementary Information [file 41467_2023_41799_MOESM1_ESM.docx]

**Supplementary Information for**

**Secondary structures that regulate mRNA translation provide insights for ASO-mediated inhibition of cardiac hypertrophy**

Omar M. Hedaya, Kadiam C. Venkata Subbaiah, Feng Jiang, Li Huitong Xie, Jiangbin Wu, EngSoon Khor, Mingyi Zhu, David H. Mathews, Chris Proschel, Peng Yao

Correspondence to Peng Yao

E-mail: [peng_yao@urmc.rochester.edu](mailto:peng_yao@urmc.rochester.edu)

**This document file includes:**

Supplementary Figures 1 to 6

SI Methods

SI References

**Supplemental figures**

**
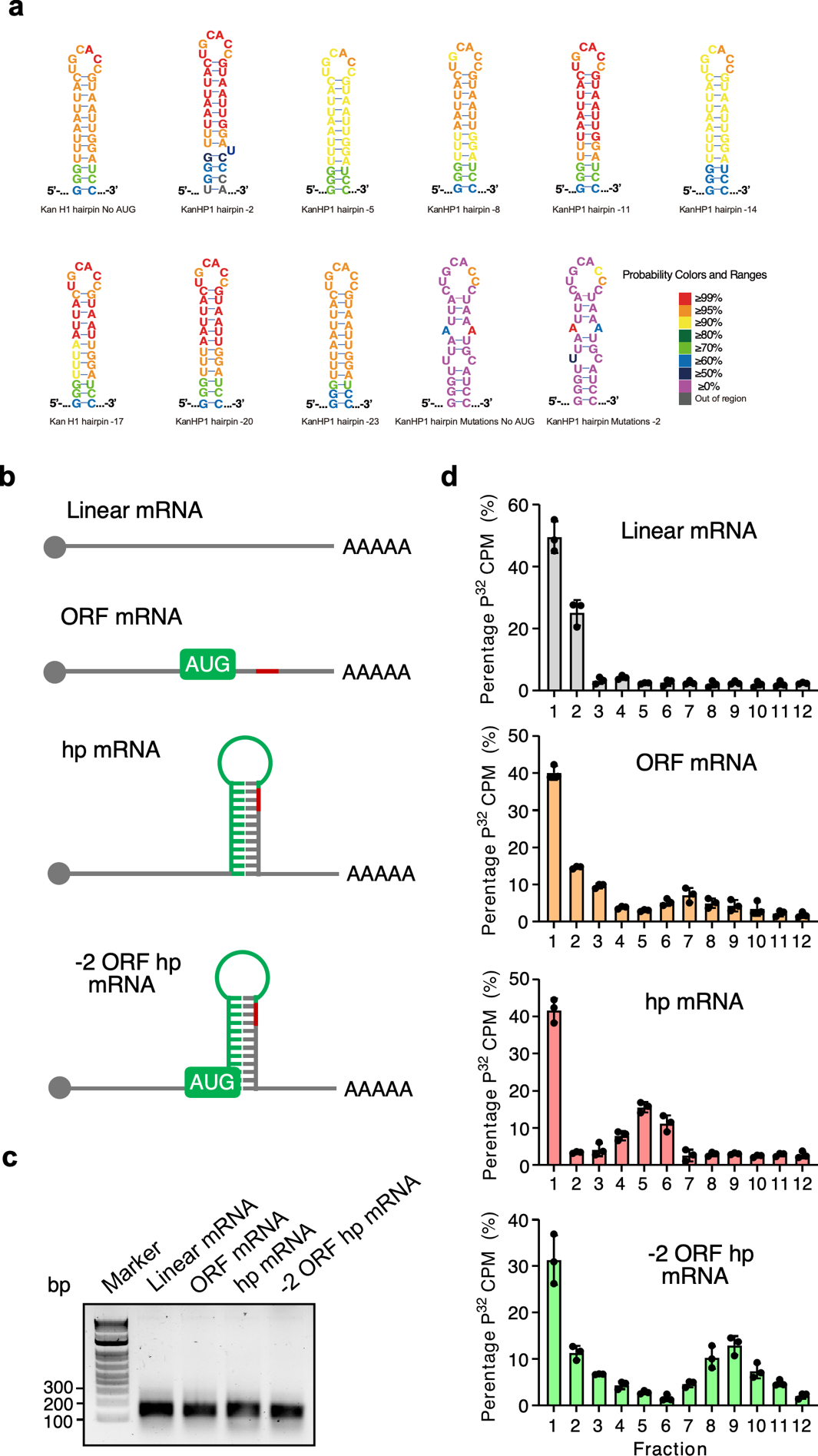
**

**Supplementary Fig. 1** **In vitro translation of uORF-KanHP1 hp and its variant mRNAs. a** Base pairing probability for the KanHP1 hairpin and negative controls. We estimated base pairing probabilities for the KanHP1 hairpin using the complete mRNA sequence to test whether it would form with high fidelity across positions.  Nucleotides in base pairs are color annotated by pairing probability, and nucleotides in loops are color annotated by the probability of being unpaired.  Color ranges are indicated by the legend. The base pairing probabilities are high for the non-mutated hairpin, showing that there is little propensity for the nucleotides to pair other than in the expected hairpin.  Additionally, the two mutant sequences (the negative controls) show low base pairing probabilities (<50%), supporting that the mutations disrupted hairpin formation. Out of region: a nucleotide not in the expected stem-loop structure. **b** Schematic model of variant mRNAs used in the rabbit reticulocyte lysate *in vitro* translation system. **c** Homogeneous and stable mRNAs were generated by in vitro transcription reactions and examined by 1% agarose gel with DNA markers. mRNAs used in our in vitro translation assays are pure and do not contain degradation products. **d** Localization of artificial *uORF-KanHP1* *hp* mRNA variants in 40S ribosomal subunit or 80S monosome fractions in rabbit reticulocyte lysates upon 10-35% sucrose gradient centrifugation. Experiments were repeated three times. Data are represented as mean ± SD (N=3 biological replicates). Source data are provided as a Source Data file.

**
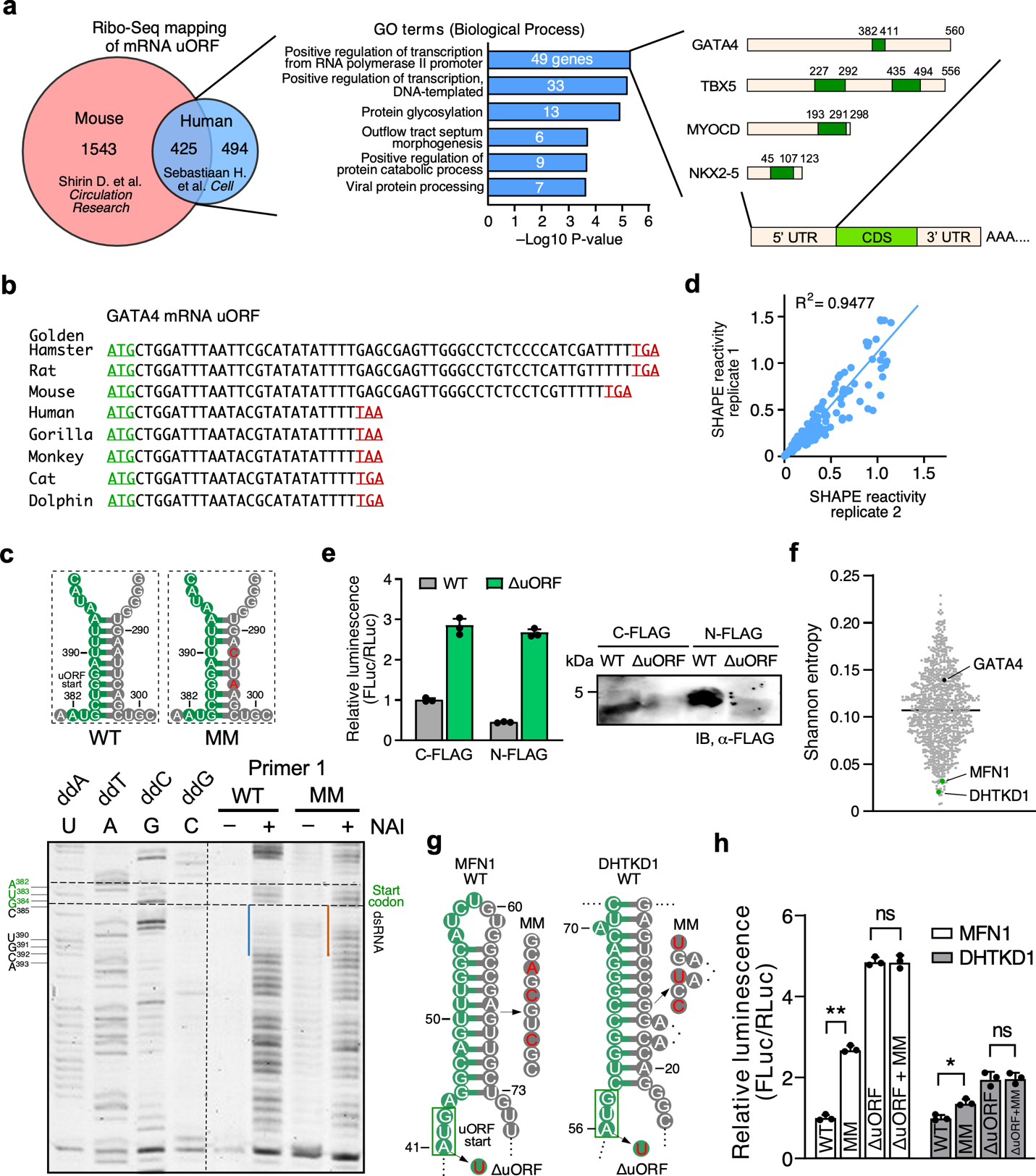
**

**
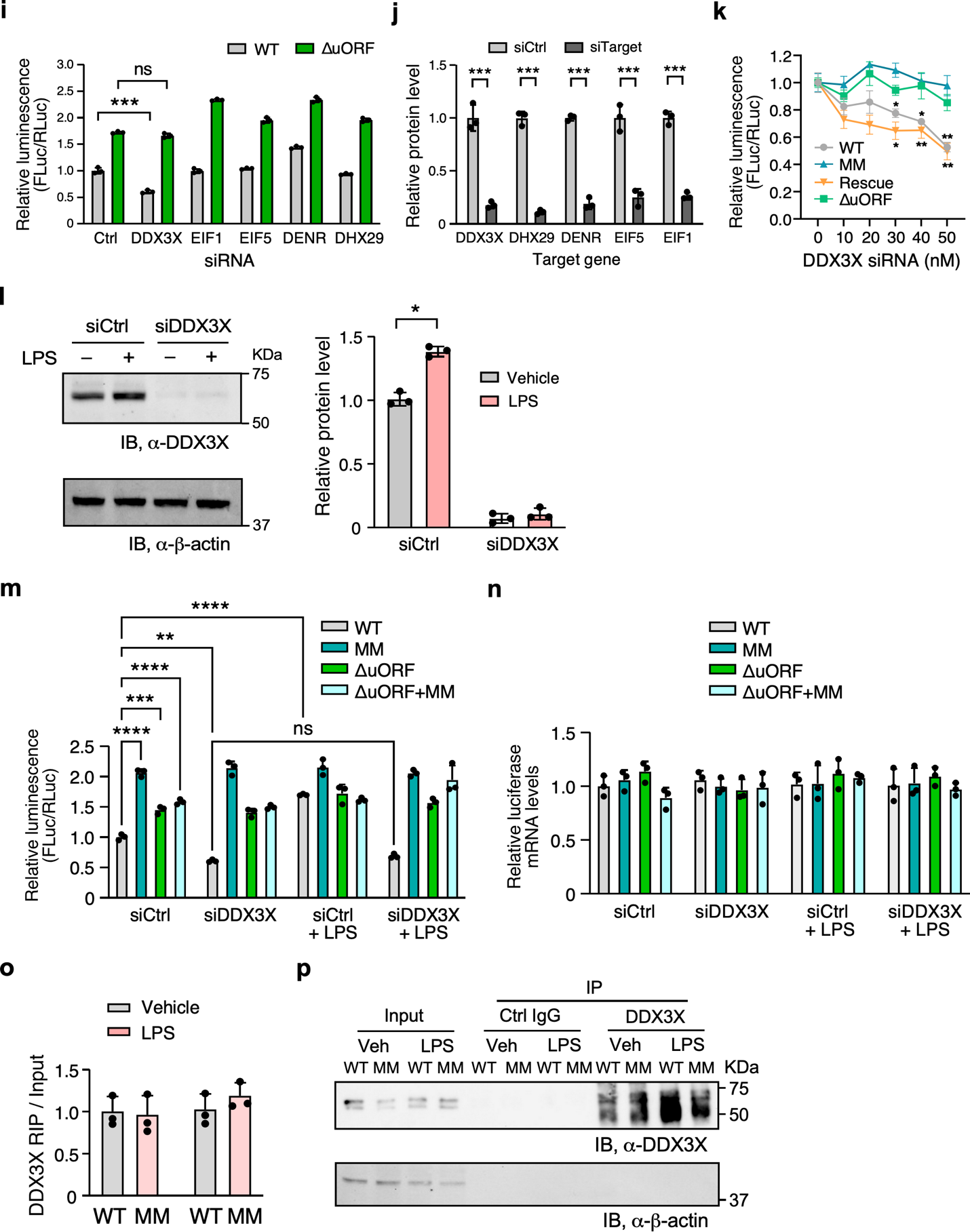
**

**Supplementary Fig. 2 Multiple mRNA transcripts contain uORF-dsRNA elements and RNA helicase DDX3X regulates *GATA4* uORF-dsRNA activity. a** Overlap of mRNAs containing uORFs based on ribosome profiling (Ribo-Seq) in human and mouse hearts. Gene ontology analysis was performed in the human cardiac uORFs. Multiple cardiac mRNAs and embedded uORFs are highlighted, such as GATA4. **b** GATA4 uORFs are present across multiple mammals, as shown in a representative species group. **c** *In vitro* RNA SHAPE analysis of the secondary structure of wild-type (WT) and mismatch (MM) mutant GATA4 uORF-dsRNA region. Primers 1 and 2 were used to detect the downstream and upstream dsRNA strands in the *GATA4* 5' UTR, respectively. **d** Pearson correlation analysis of two biological replicates of the SHAPE analysis (R-Squared value is shown). **e** Dual luciferase reporter assay with N- and C-terminal FLAG-tagged WT and ΔuORF FLuc reporters in HEK293T cells (left panel), followed by Western blot analysis of uORF peptide expression (right panel). **f** Shannon entropy was used to screen for potential uORF-dsRNA-bearing mRNAs revealed by the Ribo-seq analysis in human hearts. We focused on the sequences with the lowest Shannon entropies for subsequent experimental testing (*MFN1* and *DHKD1* are both in the lowest 2.5 percentile). **g** The predicted dsRNA structure is downstream of *MFN1* and *DHTKD1* uORF start codon by RNAstructure software. **h** Dual luciferase reporter assay with WT, ΔuORF, secondary structure mismatch (MM) mutant, and double mutants in HEK293T cells. **i** Dual luciferase reporter assay of WT and ΔuORF GATA4 5' UTR after knockdown of DDX3X, DHX29, EIF1, EIF5, and DENR in HEK293T cells. N=3 biological replicates. **j** siRNA knockdown efficiency for the candidate trans-acting factors involved in regulating dsRNA stability or uORF-mRNA translation balance. **k** Knockdown of DDX3X by incremental increase of DDX3X siRNA to test the dependance of the GATA4 5' UTR with dsRNA mismatches and rescuing mutations with or without the uORF (ΔuORF) in regulating mORF translation using dual luciferase assays in HEK293T cells. The relative luciferase activity was separately normalized to the 0 nM group for each reporter. **l** Dual luciferase reporter assay was used to measure the effect of lipopolysaccharide (LPS) treatment on the translational regulatory effect of the WT *GATA4* 5' UTR compared to one with a mismatch in the dsRNA (MM) alone or in combination with ΔuORF mutation at normal (siCtrl) or under conditions of DDX3X knockdown (siDDX3X) in HEK293T cells. **m** Relative *FLuc* mRNA does not change in (**l**) as normalized by *RLuc* mRNA. **n** Western blot measuring the protein levels of DDX3X following LPS treatment after control or DDX3X-specific siRNA treatment. **o** The MM mutation does not affect DDX3X binding to the FLuc reporter mRNA as measured using RNA-binding protein immunoprecipitation (RIP). **p** Immunoprecipitation of DDX3X or pre-immune IgG in cells expressing WT or MM mutant FLuc mRNA under treatment of LPS or vehicle. Data are represented as mean ± SD. * *P* < 0.05, ** *P* < 0.01, *** *P* < 0.001, **** *P* < 0.0001; Statistical significance was confirmed by an unpaired two-tailed Student *t* test for **h-m** (N=3 biological replicates). Source data are provided as a Source Data file.

**
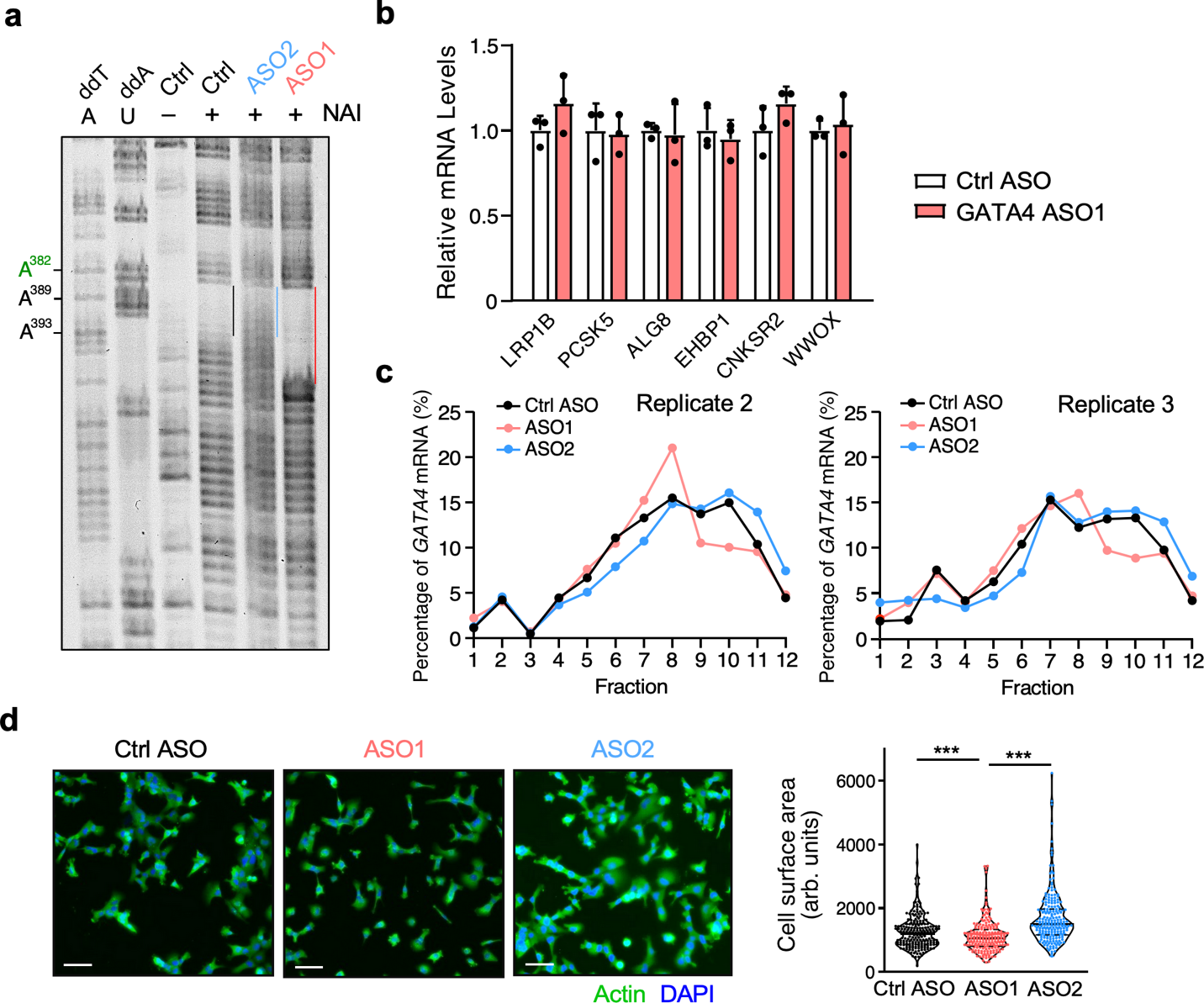
**

**Supplementary Fig. 3 *In vitro* and *in vivo* effects of GATA4 uORF-targeting ASO1 and ASO2. a** *In vitro* RNA SHAPE analysis of the secondary structure of *GATA4* uORF-dsRNA region under ASO1 and ASO2 treatment. **b** RT-qPCR measurement of putative off-target mRNAs for *GATA4* ASO1. **c** RT-qPCR measurement of *GATA4* mRNA distributions across various fractions in polysome profiles. Two biological replicates data were shown. **d** β-actin immunostaining of AC16 cells after transfection of control ASO, ASO1, and ASO2. Cell surface area was measured and quantified (n > 200 cells). Scale bar: 50 μm. Ctrl ASO is mismatched oligos. Data are represented as mean ± SD. *** *P* < 0.001; Statistical significance was confirmed by an unpaired two-tailed Student *t* test for **d**. Source data are provided as a Source Data file.

**
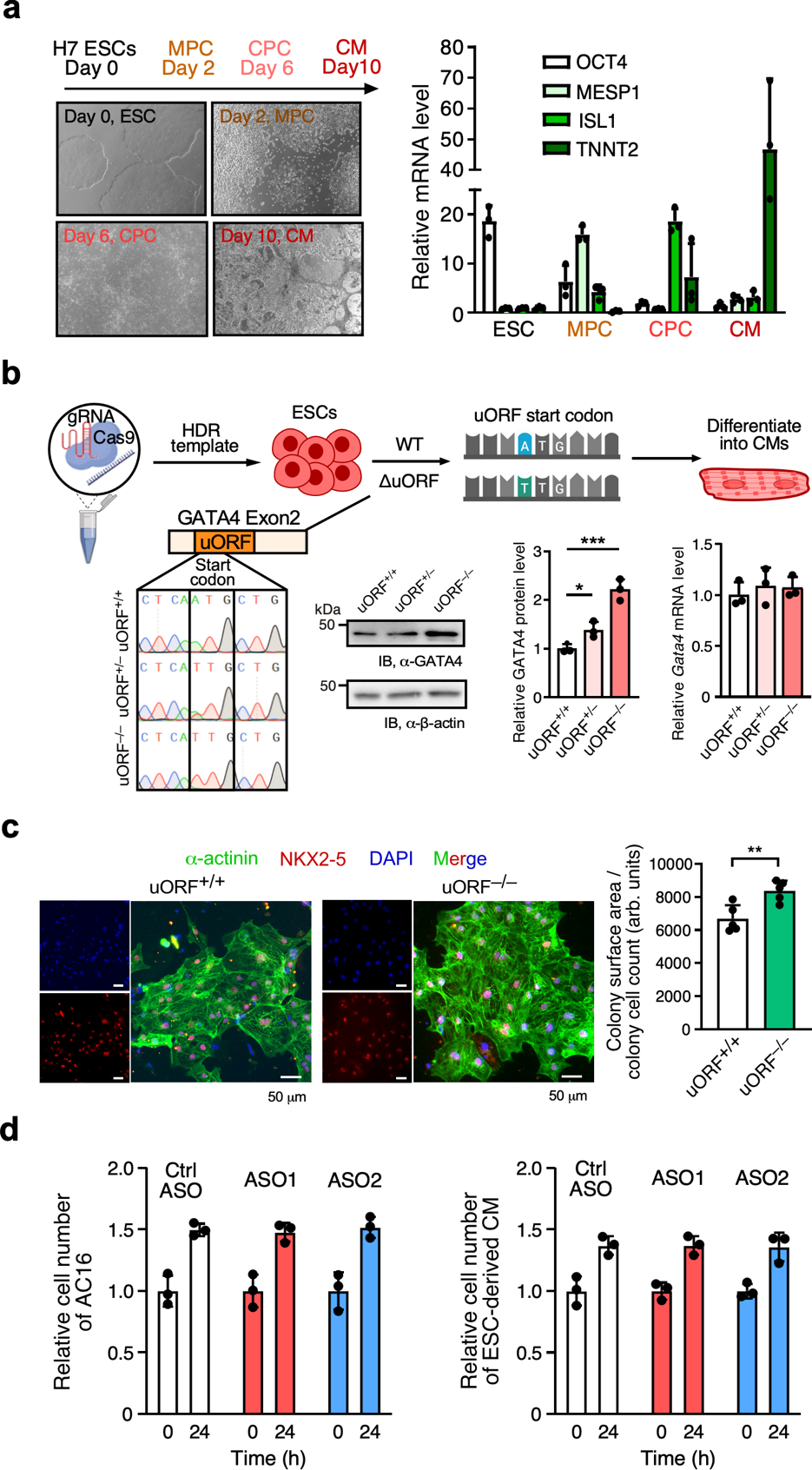
**

**Supplementary Fig. 4 Genetic inactivation of uORF increases GATA4 protein expression and CM hypertrophy in human ESC-derived CMs.** **a** Left: Human H7 ESC-to-CM differentiation timeline showing the conversion of embryonic stem cells (ESCs) to mesenchymal progenitor cells (MPCs), cardiac progenitor cells (CPCs), and finally cardiomyocytes (CMs). Right: RT-qPCR measurement of marker gene expression mRNA of *OCT4*, *MESP1*, *ISL1*, and *TNNT2*, corresponding to ESC, MPC, CPC, and CM (normalized to *ACTB* mRNA). **b** Schematic and electropherogram of CRISPR-Cas9-mediated genomic inactivation of *GATA4* uORF through ATG-to-TTG ΔuORF mutation in human H7 ESC cell line. Sanger DNA sequencing results confirmed A-to-T knock-in mutation that changed the ATG start codon to TTG. Western blotting analysis showed increased GATA4 protein expression in ΔuORF cells in a copy-dependent manner. GATA4 protein expression was normalized to β-actin. RT-qPCR showed that *GATA4* mRNA was unchanged following uORF inactivation relative to *ACTB* mRNA levels. N=3 biological replicates. **c** Left: Representative images of α-actinin (green) and NKX2-5 (red) immunostaining in addition to DAPI (blue) in ESC-derived CMs of control WT and homozygous ΔuORF mutant. Scale bar: 50 μm. Co-staining of α-actinin (green) and NKX2-5 (red) discerns CM from mis-differentiated cells. Right: Cell surface area was measured for five different clumps of cells as the total surface area divided by the number of cells (n > 100). **d** Effects of ASOs on cell proliferation in AC16 cells (left) and ESC-derived CMs (right). Data are represented as mean ± SD. * *P* < 0.05, ** *P* < 0.01, *** *P* < 0.001; Statistical significance was confirmed by an unpaired two-tailed Student *t* test for **b, c**. Source data are provided as a Source Data file.

**
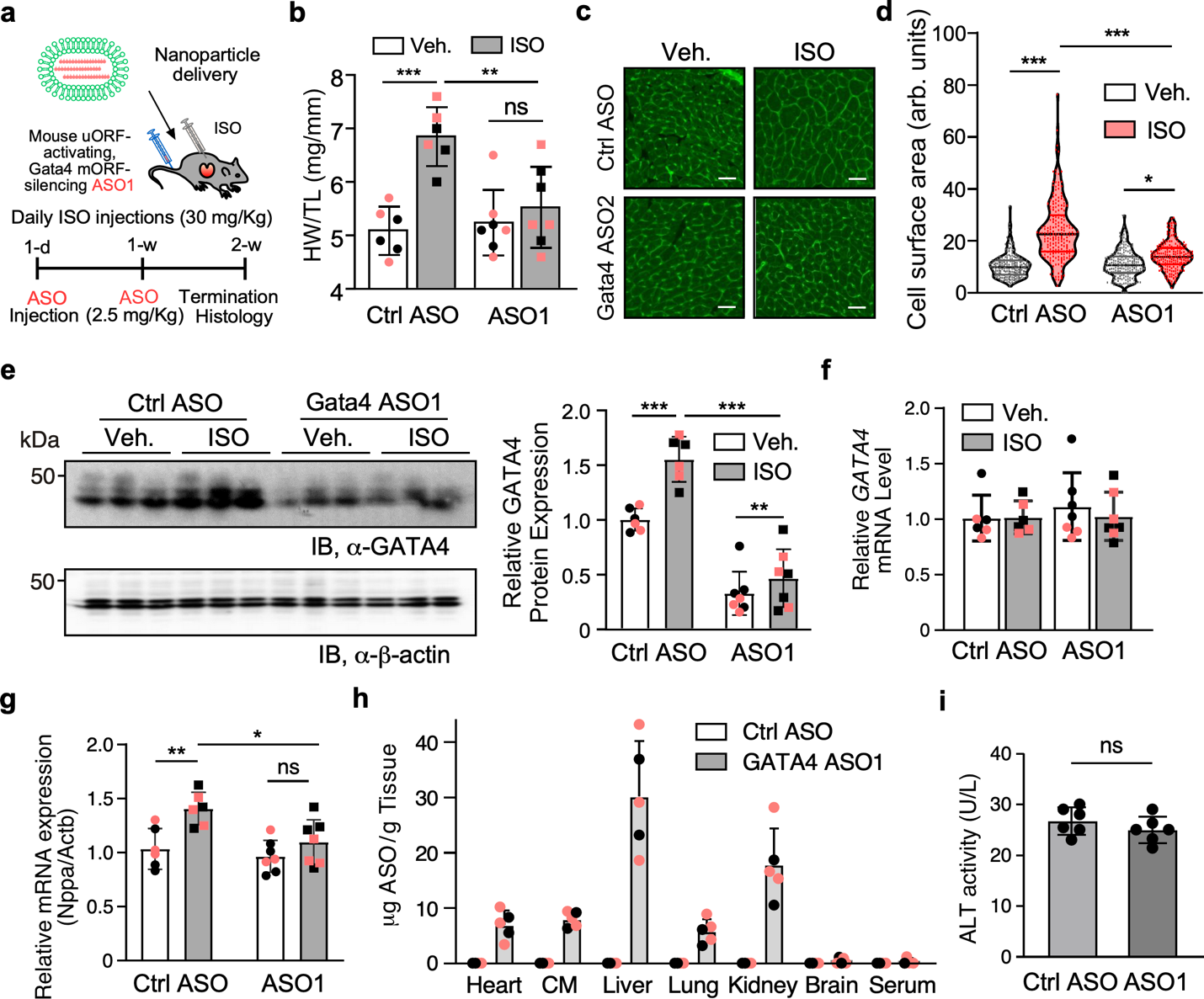
**

**Supplementary Fig. 5 Treatment of ISO-induced cardiac hypertrophy model using GATA4 ASO1.** **a** Schematic of ASO treatment of ISO-induced cardiac hypertrophy mouse model with tail-vein-injected nanoparticle-encapsulated control ASO or Gata4 ASO1. Heart samples were harvested and analyzed. Veh.-Ctrl ASO and ISO-Ctrl ASO: N=6; Veh.-Gata4 ASO and ISO-Gata4 ASO groups: N=7. Both sexes were almost equally represented, with male and female dots depicted as black and pink, respectively (**b-g**). **b** The heart weight to tibia length (HW/TL) ratio of mouse hearts in different treatment groups. **c** Representative images of wheat germ agglutinin-fluorescein in mouse heart transverse sections highlighting CM perimeter. Scale bar: 50 μm. **d** Quantification of CM cell size in (**c**). **e** Western blot analysis of GATA4 protein expression in the hearts. **f** RT-qPCR measurement of *Gata4* mRNA expression in the hearts. **g** RT-qPCR measurement of hypertrophy marker gene *Nppa* in mouse heart samples. **h** Quantification of ASO delivered to multiple organs in mice. An ASO standard curve was used for the calculation. **i** ALT activity measurement for serum from mice injected with control (Ctrl) or Gata4-specific ASOs once a week for 8 weeks. Data are represented as mean ± SD. * *P* < 0.05, ** *P* < 0.01, *** *P* < 0.001; Statistical significance was confirmed by an unpaired two-tailed Student *t* test for **i** and 2-way ANOVA followed by Holm-Sidak post hoc test for **b**, **d**-**g**. Source data are provided as a Source Data file.

**
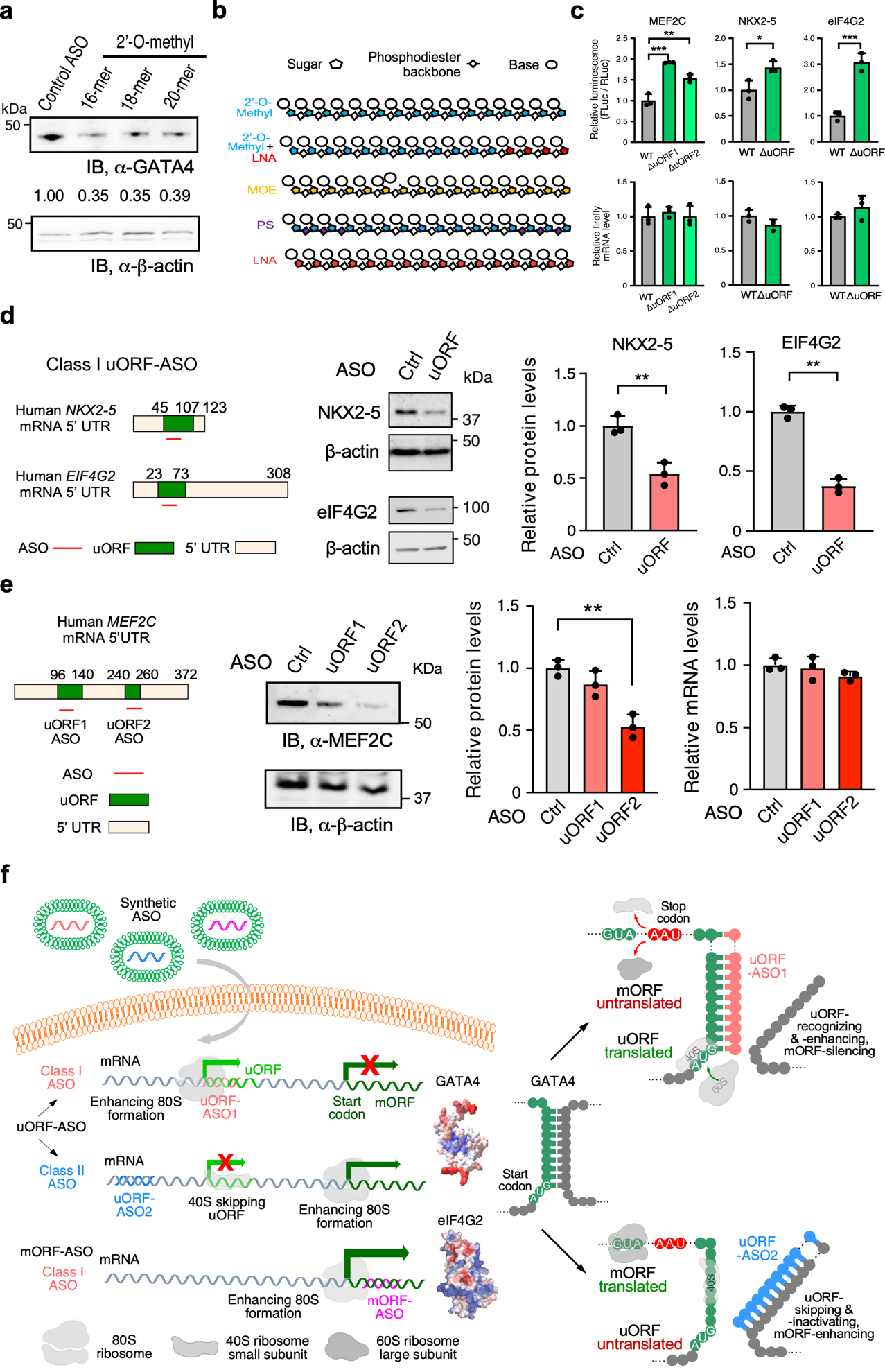
**

**Supplementary Fig. 6 Generalization of uORF- and mORF-targeted ASOs for other mRNAs encoding transcription and translation factors. a** The translation-inhibiting effects of different lengths of 2'-*O*-methyl modified GATA4 uORF-enhancing ASO1 in AC16 cells transfected with 50 nM ASO for 24 hours. The assay was repeated twice, and representative data were shown. **b** The schematic of various ASOs with different chemical modifications used in this study. **c** Top: Dual luciferase reporter assay with WT and ΔuORF mutant for MEF2C, NKX2-5, and eIF4G2 (in HEK293T cells). Bottom: RT-qPCR measurement of *FLuc* mRNAs containing *MEF2C*, *NKX2-5*, and *eIF4G2* 5' UTR. *ACTB* mRNA was used as a normalizer. **d** Western blot analysis of the three target proteins upon transfection of 50 nM uORF-enhancing ASOs (uORF-ASOs) with modifications for mRNAs of *MEF2C*, *NKX2-5*, and *eIF4G2*. **e** Western blot showing the effect of the Class I ASOs targeting MEF2C uORF1 and uORF2. The data was quantified in the right panel. We observed no significant changes in mRNA levels following ASO treatment. **f** Left: A summary model of the mechanism-based design of GATA4-inhibitory ASO for treatment of cardiac hypertrophy and the general concept of manipulating uORF activity for bidirectional control of mORF translation and direct targeting of mORF for translational activation using ASOs. Right: Schematic model of Class I uORF-ASOs activating uORF translation (top) and Class II uORF-ASOs inhibiting uORF translation (bottom) using *GATA4* as an archetypal model. Data are represented as mean ± SD. * *P* < 0.05, ** *P* < 0.01, *** *P* < 0.001. Statistical significance was confirmed by an unpaired two-tailed Student *t* test for **c-e** (N=3 biological replicates). Source data are provided as a Source Data file.

**Supplementary Methods**

**Reagents, antibodies, plasmids, and siRNAs**

Sequences of ASOs used in this study (5'-3'; “m” indicates a 2'-O-methyl modification, “e” indicates a 2'-*O*-methoxyethyl modification, “+” indicates LNA, “s” indicates phosphorothioate, and “o” indicates a phosphodiester internucleoside linkage). GATA4 human ASO control: used in humans and mice as a control; underline: mismatch compared to GATA4 ASO2. All the ASOs were synthesized in IDT, inc. 100 nmoles, purified using a desalting column. Analytical ESI-MS confirmed the purity and quality of the ASOs. Sequence information is included in the Supplementary Data 5. Antibody information is described in the Supplementary Data 6. The remaining reagent information is listed in the Supplementary Data 7.

**Animal work**

C57BL/6J mice of the same age (10-12 weeks) and gender (male and female) from littermates or sibling mating were used for experiments with WT mice. All animal procedures were performed in accordance with the National Institutes of Health (NIH) and the University of Rochester Institutional guidelines. The University of Rochester Medical Center Animal Care and Use of Committee approved all experimental animal procedures. This study used wild-type (WT) C57BL/6J mice (Jackson Laboratories). Mice were maintained on a 12-hour light/dark cycle and fed with a normal chow diet and water at 22°C with 40-60% humidity in a vivarium facility. This study used two mouse heart failure models: isoproterenol (ISO) injection and transverse aortic constriction (TAC) surgery. We used age-matched male and female mice in the study at ~8-12 weeks. All the mouse surgeries were done by the mouse Microsurgical Core facility at URMC. The mice were randomized for experiments using simple randomization with a specific ID number before animal procedures. All animal operations, including ISO injection, TAC surgery, and echocardiography measurement, were performed by the Microsurgical Core surgeons. The Histology Core did sections and histology analysis. The technicians from both Microsurgical Core and Histology Core were all blinded to the genotypes of the mice and tissue samples. For group size justification, we performed the power analysis using both G*power ^1^ version 3.1.9.6 and the function of power.anova.test in R version 3.5.3 (R Foundation for Statistical Computing, Vienna, Austria). The assumptions include the same standard variance in each study group, effect size=$\frac{Difference of the means between study groups}{common standard deviation}$, alpha level=0.05, power=0.9, and the number of study groups. The effect size for specific experiments was assumed based on similar studies or literature. In previous experiences from our Microsurgical Core, we observed a survival rate of ~90% after the TAC procedure. We added at least one mouse per treatment group to offset the possible loss of mice per treatment group.

**Isoproterenol (ISO) injection model**

Experimental mice were siblings generated from intercrosses of WT C57BL/6J mice. Age-matched WT male and female mice at 8-12 weeks of age were subjected to a vehicle (saline) or ISO treatment. ISO or vehicle saline was administered to WT mice daily for 2 weeks using subcutaneous injection (30 mg/Kg/day). At the endpoint, mice were heparinized and anesthetized via intraperitoneal injection of ketamine/xylazine. Hearts were then perfused with saline to remove the blood. Once excised, the hearts were divided into three sections. The apex and base were frozen in liquid nitrogen and kept for protein and mRNA extraction. Some tissues were fixed in 10% formalin and used for histological analyses.

**Transverse aortic constriction (TAC) surgical model**

Age-matched male and female WT C57BL/6J mice were subjected to Sham or TAC surgery at 8-12 weeks of age. Each mouse was anesthetized using 2.0% isoflurane, placed on a surgical board with a heating pad (half-inch plexiglass between the animal and the heating pad), and given buprenorphine SQ. A midline cervical incision was made to expose the trachea for visualizing oral intubation using a 22-gauge (PE90) plastic catheter. The catheter was connected to a volume-cycled ventilator supplying supplemental oxygen with a tidal volume of 225-250 μl and a respiratory rate of 120-130 strokes/min. Surgical plane anesthesia is subsequently maintained with 1-1.5% isoflurane. Procedure for left thoracotomy: Skin was incised, and the chest cavity was opened at the level of the 2^nd^ intercostal space. The transverse section of the aorta was isolated. Transverse aortic constriction was created by securely placing a (6-0 silk) ligature around the trans-aorta and a 27-gauge needle, causing complete occlusion of the aorta. The needle was removed, restoring a lumen with severe stenosis. Lungs were reinflated, and the chest was closed using Vicryl 6-0 suture. Muscle and skin were sutured using a Vicryl 6-0 suture in a running subcuticular pattern. Once the mouse was breathing on its own, it was removed from the ventilator and allowed to recover in a clean cage on a heated pad.

**Measurement of alanine aminotransferase (ALT) activity**

The alanine transaminase activity was measured using the ALT assay kit, based on a colorimetric method. Isolated tissues were homogenized in 100 mM Tris-HCl buffer (pH 7.8), and centrifuged at 10,000 g for 15 min. The ALT substrate and cofactors were added to the diluted supernatant in the 96-well ELISA plate and incubated at 37 °C for 15 min. Then the reaction was started by adding the ALT initiator and measured at 340 nm. The activity is represented as U/L.

**CRISPR genomic editing in human embryonic stem cells**

The gRNA sequences were designed based on the online database CHOPCHOP^2^ and UCSC genome browser track “CRISPR targets”^3,4^. The single-stranded oligodeoxynucleotides (ssODNs) templates for homologous repair were designed by flanking the edition sites with 30 nt homologous arms at both sides. Both gRNAs and ssODNs were synthesized by IDT. According to the manufacturer's instructions, the gRNA-Cas9 RNA-protein complex was delivered into PSCs through the Neon transfection system (Thermo Fisher Scientific). Briefly, 10 pmol gRNA was mixed with 1.5 μg TrueCut Cas9 in 15 μL Buffer R. The mixture was incubated for 15 min at room temperature to allow the assembly of the Cas9-gRNA complex, then 4.5 pmol ssODNs and 15 pmol electroporation enhancer (IDT) were added to the mixture. To dissociate the ESCs into single cells, the colonies were washed with PBS and incubated at 37˚C for 2 min with 50% TrypLE-select diluted in PBS. The dissociated cells were counted, and 12,000 cells were collected for each electroporation. The cells were washed twice with PBS and resuspended with a 10 μL Cas9-gRNA mixture for electroporation. The setting for Cas9 electroporation in human ESCs was a single 30 ms pulse at 1200 V. The transfected cells were then plated on the iMatrix511-coated surface in StemFlex medium supplemented with ROCK inhibitor Y27632 (5 μM). The single cell-derived colonies were allowed to expand for 6 days and were then manually collected for further passaging and validation. Polymerase chain reaction (PCR) and Sanger sequencing were performed to identify colonies with correct genome editing.

gRNA (Alt-R CRISPR-Cas9 sgRNA; IDT):

mU*mA*mG*rArGrCrCrCrUrUrUrGrCrUrCrArArUrGrCrGrUrUrUrUrArGrArGrCrUrArGrArArArUrArGrCrArArGrUrUrArArArArUrArArGrGrCrUrArGrUrCrCrGrUrUrArUrCrArArCrUrUrGrArArArArArGrUrGrGrCrArCrCrGrArGrUrCrGrGrUrGrCmU*mU*mU*rU

ssODN: C*C*G*GAGTAAACAAGAGCCTAGAGCCCTTTGCTCATTGCTGGATTTAATACGTATATATTTTTAAGCGAGTTGGTTT*T*T*T

*: phosphorothioate backbone

**Embryonic stem cell (ESC)-to-cardiomyocyte (CM) differentiation system**

The generation of CMs from human ESCs was achieved using the PSC Cardiomyocyte Differentiation Kit, following the manufacturer’s instructions. Briefly, the H7 ESC colonies were dissociated into single cells using TrypLE select. Around 80,000 cells were resuspended with 2 ml StemFlex medium supplemented with ROCK inhibitor seeded on each well of Geltrex-coated 6-well plates. The cells were maintained in StemFlex medium until they reached 70-80% confluence. Then, the medium was exchanged to Cardiomyocyte Differentiation Medium A on Day 1, Cardiomyocyte Differentiation Medium B on Day 3, and Cardiomyocyte Maintenance Medium on Day 5 and after. The complete medium change was performed every other day. On Day 11, the differentiated cells were dissociated with TrypLE select and seeded on a Geltrex-coated surface for further analysis. Throughout the differentiation, the cells were cultured at 37˚C with 5% oxygen and 7.5% CO_2_.

**Dual-luciferase reporter assay**

Transfected cells were incubated with Dual-Glo luciferase substrate according to the manufacturer’s recommendations. The final readings of the FLuc were normalized to RLuc to obtain the relative luminescence reading. Folding free energy changes for hairpin stem-loops were estimated using the efn2 program in RNAstructure with default settings.  The folding free energy changes include the terminal mismatches at the base of the stem, which are known to stabilize folding.

Sequence information of FLuc reporters in Fig. 1 (BsmB1 sites; **CA-rich linear sequence**; **KanHP1 hairpin**; **ATG or TAA**; **inserted CA linear sequence**; **MM mutations**):

No AUG:

AACGTCTCC**ACACCACACACACACACACACACACACACACACACACACACACAGGGTTTAATTACTGCACCGTAATTGGATCCCACACACACACACACACACATG**GAAGAGAGACGTT

-2:

AACGTCTCC**ACACCACACACACACACACACACACACACACACACACACACACATGGGTTTAATTACTGCACCGTAATTGGATCCCACACACACACACACACACATG**GAAGAGAGACGTT

-5:

AACGTCTCC**ACACCACACACACACACACACACACACACACACACACACAUGCAGGGTTTAATTACTGCACCGTAATTGGATCCCACACACACACACACACACATG**GAAGAGAGACGTT

-8:

AACGTCTCC**ACACCACACACACACACACACACACACACACACACAAUGACACAGGGTTTAATTACTGCACCGTAATTGGATCCCACACACACACACACACACATG**GAAGAGAGACGTT

-11:

AACGTCTCC**ACACCACACACACACACACACACACACACACACAUGCACACACAGGGTTTAATTACTGCACCGTAATTGGATCCCACACACACACACACACACATG**GAAGAGAGACGTT

-14:

AACGTCTCC**ACACCACACACACACACACACACACACACAAUGACACACACACAGGGTTTAATTACTGCACCGTAATTGGATCCCACACACACACACACACACATG**GAAGAGAGACGTT

-17:

AACGTCTCC**ACACCACACACACACACACACACACACAUGCACACACACACACAGGGTTTAATTACTGCACCGTAATTGGATCCCACACACACACACACACACATG**GAAGAGAGACGTT

-20:

AACGTCTCC**ACACCACACACACACACACACACAAUGACACACACACACACACAGGGTTTAATTACTGCACCGTAATTGGATCCCACACACACACACACACACATG**GAAGAGAGACGTT

-23:

AACGTCTCC**ACACCACACACACACACACACAUGCACACACACACACACACACAGGGTTTAATTACTGCACCGTAATTGGATCCCACACACACACACACACACATG**GAAGAGAGACGTT

-2 MM:

AACGTCTCC**ACACCACACACACACACACACACACACACACACACACACACACATGGGTTTAATTACTGCACCCTAAATGCATCCCACACACACACACACACACATG**GAAGAGAGACGTT

**In vitro ribosome loading and sucrose gradient fractionation in RRL**

To explore the mechanism underlying the dsRNA-mediated influence of uORF translation on mORF translation, we internally labeled four *in vitro*-transcribed mRNAs using the [α-^32^P]-ATP (250 mCi) and added poly(A) tails for the sequences below using HiScribe™ T7 ARCA mRNA Kit (with tailing). The RNA was extracted as described in RNA SHAPE. Each RNA (5 μl) was added to rabbit reticulocyte lysate reactions according to the manufacturer’s recommendations and incubated for 30 min at 30°C. The whole lysate was added to 15% sucrose gradients and centrifuged at 150,000g for 2 hours and 20 min. The gradients were fractionated by hand into 12 tubes. The radioactivity was quantified using liquid scintillation for each tube. The profile for an individual mRNA indicates the fraction of the sum of the total radioactivity across each tube.

Sequence information of 5' UTR RNA used in Supplementary Fig. 1 (KanHP1 hairpin):

T7 promoter (TAATACGACTCACTATAG**)** +

Linear mRNA:

CACACCACACACACACACACACACACACACACACACACACACACAcacacacacacacacacacacacacacacaCACACACACACACACACAC

ORF mRNA (ATG is the start codon, and TAA is the stop codon):

CACACCACACACACACACACACACACACACACACACACACACACATGcacacacacacacacacaTAAcacacacacACACACACACACACACAC

hp mRNA: (hp is highlighted in blue)

CACACCACACACACACACACACACACACACACACACACACACACA**GGGTTTAATTACTGCACCGTAATTGGATCC**CACACACACACACACACAC

-2 ORF hp mRNA:

CACACCACACACACACACACACACACACACACACACACACACACAT**GGGTTTAATTACTGCACCGTAATTGGATCC**CACACACACACACACACAC

**Selective 2' Hydroxyl Acylation analyzed by Primer Extension (SHAPE) assay**

The following sequence of 257-nt (209-465 nt) wild-type GATA4 5' UTR RNA (for SHAPE): ctcgtgcgccacctccaggcctggacgctgccctccgtcttctgcccccaataggtgcgccggaccttcaggccctggggtgaattcagctgctcctacatcagcttccggaaccaccaaaaattcaaattgggattttccggagtaaacaagagcctagagccctttgctcaatgctggatttaatacgtatatatttttaagcgagttggttttttcccctttgatttttgatcttcgcgacagttcctcccacg (Blue: target site of GATA4 Primer 2; Yellow: target site of GATA4 Primer 1) was PCR amplified from the GATA4 5' UTR using the forward T7 promoter containing forward primer 5’ TAATACGACTCACTATAG CTCGTGCGCCACCTCCAG 3’ and the reverse primer 5’ CGTGGGAGGAACTGTCGCG 3’. The RNA was transcribed using HiScribe T7 Quick High Yield RNA Synthesis Kit. The RNA was purified as discussed above and reconstituted in RNase-free water. Purified RNA from in vitro transcription was heated in RNase-free water for 2 min at 95°C, then flash-cooled on ice. A 3× SHAPE buffer [0.333 M HEPES (pH 8.0), 0.02 M MgCl_2_, 0.333 M NaCl] was added, and the RNA was equilibrated at 37°C for 10 min with or without ASOs. The RNA was then incubated at 37°C for 15 min. To this mixture, 1 μL of 10× NAI (2-methylnicotinic acid imidazolide) stock in DMSO (+), or DMSO alone (−), was added to a final concentration of 25 mM. The NAI reaction proceeded for 15 min. RNA was extracted and reverse transcribed using two CY5-labelled primer sequences (in the reagent table list), and visualized using 8% UREA (8 M) PAGE^5^. The gel bands were quantified in SAFA (Semi-Automated Footprinting Analysis)^6^. The SHAPE reactivity values were cleared of outliers and normalized. Then, the final values were used as SHAPE constraints for the RNA Fold web server below^7^.

<https://rna.urmc.rochester.edu/RNAstructureWeb/Servers/Predict1/Predict1.html>

The sequence of 257-nt Mismatch (MM) GATA4 5' UTR RNA (for SHAPE):

cUcgUgcgccaccUccaggccUggacgcUgcccUccgUcUUcUgcccccaaUaggUgcgccggaccUUcaggcccUggggUgaCUUAagcUgcUccUacaUcagcUUccggaaccaccaaaaaUUcaaaUUgggaUUUUccggagUaaacaagagccUagagcccUUUgcUcaaUgcUggaUUUaaUacgUaUaUaUUUUUaagcgagUUggUUUUUUccccUUUgaUUUUUgaUcUUcgcgacagUUccUcccacg

Code for SHAPE normalization is included in the Source data file as a txt. file.

**Picrosirius red staining**

Paraffin-embedded tissue sections were deparaffinized and incubated in a picrosirius red solution at RT for 1 hour. Then, slides were subjected to 2 washes of 1% acetic acid and 100% of ethyl alcohol and mounted in a resinous medium. Images were captured using the PrimeHisto XE Histology Slide Scanner (Carolina). Six images were selected from each group for analysis. Total collagen content was determined for the whole heart images using the Image J software.

### RNA structure prediction and Shannon Entropy calculation

We quantified the propensity of sequence to fold to a single, well-ordered structure using the Shannon Entropy^8,9^, S:

$$S=-\sum_{i=i}^{N} \sum_{j=i+3}^{N} P_{i,j}\cdot\log_{10} \left( P_{i,j} \right)$$

where N is the length of the sequence and P_i,j_ is the base pairing probability estimated for the pair between nucleotides i and j. The partition program in RNAstructure (version 6.2) was used to estimate the RNA base pair probabilities^10^. The 5' UTR region of sequences were used to calculate partition function and Shannon Entropy. Shannon entropy has been used to identify functional secondary structures^11-13^.

5' UTRs were determined based on the annotations from Ensemble and Gencode.v40:

<http://ftp.ebi.ac.uk/pub/databases/gencode/Gencode_human/release_40/gencode.v40.annotation.gff3.gz>

Sequences of the 5' UTRs were retrieved from GRCh38.p13:

<https://ftp.ebi.ac.uk/pub/databases/gencode/Gencode_human/release_40/gencode.v40.transcripts.fa.gz>)

**Supplementary References**

1 Faul, F., Erdfelder, E., Lang, A. G. & Buchner, A. G*Power 3: a flexible statistical power analysis program for the social, behavioral, and biomedical sciences. *Behav Res Methods* **39**, 175-191, doi:10.3758/bf03193146 (2007).

2 Labun, K. *et al.* CHOPCHOP v3: expanding the CRISPR web toolbox beyond genome editing. *Nucleic Acids Res* **47**, W171-W174, doi:10.1093/nar/gkz365 (2019).

3 Haeussler, M. *et al.* Evaluation of off-target and on-target scoring algorithms and integration into the guide RNA selection tool CRISPOR. *Genome Biol* **17**, 148, doi:10.1186/s13059-016-1012-2 (2016).

4 Doench, J. G. *et al.* Optimized sgRNA design to maximize activity and minimize off-target effects of CRISPR-Cas9. *Nat Biotechnol* **34**, 184-191, doi:10.1038/nbt.3437 (2016).

5 Wilkinson, K. A., Merino, E. J. & Weeks, K. M. Selective 2'-hydroxyl acylation analyzed by primer extension (SHAPE): quantitative RNA structure analysis at single nucleotide resolution. *Nat Protoc* **1**, 1610-1616, doi:10.1038/nprot.2006.249 (2006).

6 Laederach, A. *et al.* Semiautomated and rapid quantification of nucleic acid footprinting and structure mapping experiments. *Nat Protoc* **3**, 1395-1401, doi:10.1038/nprot.2008.134 (2008).

7 Bellaousov, S., Reuter, J. S., Seetin, M. G. & Mathews, D. H. RNAstructure: Web servers for RNA secondary structure prediction and analysis. *Nucleic Acids Res* **41**, W471-474, doi:10.1093/nar/gkt290 (2013).

8 Huynen, M., Gutell, R. & Konings, D. Assessing the reliability of RNA folding using statistical mechanics. *J Mol Biol* **267**, 1104-1112, doi:10.1006/jmbi.1997.0889 (1997).

9 Mathews, D. H. Using an RNA secondary structure partition function to determine confidence in base pairs predicted by free energy minimization. *RNA* **10**, 1178-1190, doi:10.1261/rna.7650904 (2004).

10 Reuter, J. S. & Mathews, D. H. RNAstructure: software for RNA secondary structure prediction and analysis. *BMC Bioinformatics* **11**, 129, doi:10.1186/1471-2105-11-129 (2010).

11 Siegfried, N. A., Busan, S., Rice, G. M., Nelson, J. A. & Weeks, K. M. RNA motif discovery by SHAPE and mutational profiling (SHAPE-MaP). *Nat Methods* **11**, 959-965, doi:10.1038/nmeth.3029 (2014).

12 Huston, N. C. *et al.* Comprehensive in vivo secondary structure of the SARS-CoV-2 genome reveals novel regulatory motifs and mechanisms. *Mol Cell* **81**, 584-598 e585, doi:10.1016/j.molcel.2020.12.041 (2021).

13 Szutkowska, B. *et al.* Secondary Structure of Influenza A Virus Genomic Segment 8 RNA Folded in a Cellular Environment. *Int J Mol Sci* **23**, doi:10.3390/ijms23052452 (2022).
