# Supplementary material for: Secondary structures that regulate mRNA translation provide insights for ASO-mediated modulation of cardiac hypertrophy
Source: Nat Commun. 2023 Oct 3;14:6166. doi: 10.1038/s41467-023-41799-1 (PMC10547706; doi:10.1038/s41467-023-41799-1)
Supplement: Supplementary file 3 — Description of Additional Supplementary Files [file 41467_2023_41799_MOESM3_ESM.pdf]

Description of Additional Supplementary Files

File Name: Supplementary Data 1

Description: The number of human mRNA 5' UTRs that contain different numbers of AUG start codons (**Fig. 1a**). Percentage of GC-rich sequences in human mRNA 5' UTR, CDS, and 3' UTR (**Fig. 1b**).

File Name: Supplementary Data 2

Description: Overlap of mRNAs containing uORFs based on ribosome profiling (Ribo-Seq) in human and mouse hearts. Gene ontology analysis was performed on the human and mouse overlapped cardiac uORF-bearing genes.

File Name: Supplementary Data 3

Description: Shannon entropy calculations for sequences upstream of the mORF of mRNAs that contain translated uORFs in human hearts.

File Name: Supplementary Data 4

Description: Echocardiographic analysis (M-mode short axis) of WT mice (male and female) up to 8 weeks post-TAC surgery compared to sham surgery.

File Name: Supplementary Data 5

Description: Sequences of ASOs, siRNA, probes for ASO quantification, and primers (for cloning, RT-qPCR, and SHAPE analysis) used in this study are listed.

File Name: Supplementary Data 6

Description: Information of antibodies used in this study, including catalog number and dilution factor.

File Name: Supplementary Data 7

Description: Information on other reagents used in this study, including cell lines, chemicals, kits, and plasmids.

File Name: Supplementary Data 8

Description: Information on mRNA sequences used in this study. NCBI accession numbers are provided.

File Name: Supplementary Data 9

Description: Code for SHAPE normalization
