# Supplementary material for: Secondary structures that regulate mRNA translation provide insights for ASO-mediated modulation of cardiac hypertrophy
Source: Nat Commun. 2023 Oct 3;14:6166. doi: 10.1038/s41467-023-41799-1 (PMC10547706; doi:10.1038/s41467-023-41799-1)
Supplement: Supplementary file 11 — Supplementary Data 8 [file 41467_2023_41799_MOESM11_ESM.pdf]

Human *GATA4* mRNA: NM_002052.5

uORF sequence: 382-411 (30-nt)

mORF sequence: 561-1892 (1332-nt)

uORF-ASO1 target sequence

uORF-ASO2 target sequence

mORF-ASO target sequence

```
   1 ggggacuugg aggcggccgg cgcaggggcc gcgagaggcu ucgucgccgc ugcagcuccg
  61 ggggcuccca ggggagcgug cgcggaaccu ccaggcccag caggaccccg gcugcggcga
 121 ggaggaagga gccagccuag cagcuucugc gccuguggcc gcgggugucc uggaggccuc
 181 ucggugugac gaguggggga cccgaaggcu cgugcgccac cuccaggccu ggacgcugcc
 241 cuccgucuuc ugcccccaau aggugcgccg gaccuucagg cccuggggug aauucagcug
 301 cuccuacauc agcuuccgga accaccaaaa auucaaauug ggauuuuccg gaguaaacaa
 361 gagccuagag cccuuugcuc aaugcuggau uuaauacgua uauauuuuua agcgaguugg
 421 uuuuuucccc uuugauuuuu gaucuucgcg acaguuccuc ccacgcauau uaucguuguu
 481 gccgucguuu ucucuccccg cguggcuccu ugaccugcga gggagagaga ggacaccgaa
 541 gccgggagcu cgcagggacc auguaucaga gcuuggccau ggccgccaac cacgggccgc
 601 cccccggugc cuacgaggcg ggcggccccg gcgccuucau gcacggcgcg ggcgccgcgu
 661 ccucgccagu cuacgugccc acaccgcggg ugcccuccuc cgugcugggc cuguccuacc
 721 uccagggcgg aggcgcgggc ucugcguccg gaggcgccuc gggcggcagc uccggugggg
 781 ccgcgucugg ugcggggccc gggacccagc agggcagccc gggauggagc caggcgggag
 841 ccgacggagc cgcuuacacc ccgccgccgg ugucgccgcg cuucuccuuc ccggggacca
 901 ccggguccu ggcggccgcc gccgccgcug ccgcggcccg ggaagcugcg gccuacagca
 961 guggcggcgg agcggcgggu gcgggccugg cgggccgcga gcaguacggg cgcgccggcu
1021 ucgcgggcuc cuacuccagc cccuacccgg cuuacauggc cgacgugggc gcguccuggg
1081 ccgcagccgc cgccgccucc gccggcccu ucgacagccc gguccugcac agccugcccg
1141 gccgggccaa cccggccgcc cgacacccca aucucguaga uauguuugac gacuucucag
```

1201 aaggcagaga gugugucaac uguggggcua uguccacccc gcucuggagg cgagauggga

1261 cgggucacua ucugugcaac gccugcggcc ucuaccacaa gaugaacggc aucaaccggc

1321 cgcucaucaa gccucagcgc cggcuguccg ccucccgccg agugggccuc uccugugcca

1381 acugccagac caccaccacc acgcuguggc gccgcaaugc ggagggcgag ccugugugca

1441 augccugcgg ccucuacaug aagcuccacg gggucccag gccucuugca augcggaaag

1501 aggggaucca aaccagaaaa cggaagccca agaaccugaa uaaaucuaag acaccagcag

1561 cuccuucagg cagugagagc cuuccucccg ccagcggugc uuccagcaac uccagcaacg

1621 ccaccaccag cagcagcgag gagaugcguc ccaucaagac ggagccuggc cugucaucuc

1681 acuacgggca cagcagcucc gugucccaga cguucucagu cagugcgaug ucuggccaug

1741 ggcccuccau ccacccuguc cucucggccc ugaagcucuc cccacaaggc uaugcgucuc

1801 ccgucagcca gucuccacag accagcucca agcaggacuc uuggaacagc cuggucuugg

1861 ccgacaguca cggggacaua aucacugcgu aa 3'UTR

Mouse *Gata4* mRNA: NM_008092.4

uORF: 426-482 (57-nt)

mORF: 619-1947 (1329-nt)

uORF-ASO1 target sequence

1 gcgccgggag caggggacaa gccggaggcc cgcagagugg ccgcccgagg cucagccgca

61 guugcagcuc cgcggacuca cggagaucgc gccgguuuuc ugggaaacug gagcuggcca

121 ggacugccgc uucgcuucga agggaccggg cccucuuugu cauucuucgc uggagccgcu

181 cuggagcuag cagcugcgcc ugggugugua gcaggcagaa agcaaggacu aggcuucuuu

241 agccggugg ugauccgaag gccugcucag gguguucgag accagccugg acugcgucug

301 ggcaccucca gccucugggc ccuggaauag aguccgcccu cccgcacgau uucuggagca

361 accgcaaauc caauuuggga uuuucuuuuu ccugagcaaa ccagagccua gagguuucug

421 cuuugaugcu ggauuuaauu cguauauauu uugagcgagu ugggccucuc cucguuuuuu

481 gaucuccggu uguuuuuuuu uuggggggggg gguuaguuuu uggguuuuug uuuuguuuug

541 uuuuguuuug auuuuuggug acaguuccgc acacccgcau ucuaguucuu gucugccucg

601 ugcucagagc uuggggcgau guaccaaagc cuggccaugg ccgccaacca cggcccccccg

661 cccggcgccu acgaagcagg uggcccuggc gccuucaugc acagcgcggg cgccgcgucc

721 ucgcccgucu acgugcccac uccgcgggug ccguccucug ugcugggccu guccuaccug

781 cagggcggug gcagugccgc ugcagcugga accaccucgg guggcagcuc cggggccggc

841 ccgucgggug cagggccugg gacccagcag gguagcccug gcuggagcca agcuggagcc

901 gagggagccg ccuacacccc gccgcccgug uccccgcgcu ucucuuuccc ggggacuacu

961 ggguccccugg cggccgcugc cgccgcugcc gcagcccggg aagcugcagc cuacggcagu

1021 ggcggcgggg cggcgggcgc uggucuggcu ggccgagagc aguacgggcg uccgggcuuc

1081 gccggcuccu acuccagccc cuacccagcc uacauggccg acgugggagc auccugggcc

1141 gcagccgcug ccgccucugc cggccccuuc gacagcccag uccugcacag ccugccugga

1201 cgggccaacc cuggaagaca ccccaaucuc guagauaugu uugaugacuu cucagaaggc

1261 agagagugug ucaauugugg ggccaugucc accccacucu ggaggcgaga ugggacggga

1321 cacuaccugu gcaaugccug uggccucuau cacaagauga acggcaucaa ccggccccuc

1381 auuaagccuc agcgccgccu guccgcuucc cgccggguag gccucuccug ugccaacugc

1441 cagacuacca ccaccacgcu guggcgucgu aaugccgagg gugagccugu auguaaugcc

1501 ugcggccucu acaugaagcu ccaugggguu cccaggccuc uugcaaugcg gaaggagggg

1561 auucaaacca gaaaacggaa gcccaagaac cugaauaaau cuaagacgcc agcagguccu

1621 gcuggugaga cccucccucc cuccaguggu gccuccagcg guaacuccag caaugccacu

1681 agcagcagca gcagcaguga agagaugcgc cccaucaaga cagagcccgg gcugucaucu

1741 cacuaugggc acagcagcuc caugucccag acauucagua cuguguccgg ccacgggccc

1801 uccauccauc cagugcuguc ugcucugaag cuguccccac aaggcuaugc aucuccuguc

1861 acucagacau cgcaggccag cucccaagcag gacucuugga acagccuggu ccuggcugac

1921 agucaugggg acauaaucac cgcguaa 3'UTR

Human *MEF2C* mRNA: NM_002397.5

uORF sequence: uORF1: 96-140 (45-nt); uORF2: 240-260 (21-nt)

mORF sequence: 373-1794 (1422-nt)

uORF-ASO target sequence

mORF-ASO target sequence

```
   1 gcagucacag acacuugagc acacgcguac acccagacau cuucgggcug cuauuggauu
  61 gacuuugaag guucugugug ggucgccgug gcugcauguu ugaaucaggu ggagaagcac
 121 uucaacgcug gacgaaguaa agauuauugu uguuauuuuu uuuuucucuc ucucucucuc
 181 uuaagaaagg aaaauauccc aaggacuaau cugaucgggu cuuccuucau caggaacgaa
 241 ugcaggaauu ugggaacuga gcugugcaag ugcugaagaa ggagauuugu uuggaggaaa
 301 caggaaagag aaagaaaagg aaggaaaaaa uacauaauuu cagggacgag agagagaaga
 361 aaaacgggga cuaugggggag aaaaaagauu cagauuacga ggauuaugga ugaacguaac
 421 agacagguga cauuuacaaa gaggaaauuu ggguugauga agaaggcuua ugagcugagc
 481 gugcugugug acugugagau ugcgcugauc aucuucaaca gcaccaacaa gcuguuccag
 541 uaugccagca ccgacaugga caaagugcuu cucaaguaca cggaguacaa cgagccgcau
 601 gagagccgga caaacucaga caucguggag acguugagaa agaagggccu uaauggcugu
 661 gacagcccag accccgaugc ggacgauucc guaggucaca gcccugaguc ugaggacaag
 721 uacaggaaaa uuaacgaaga uauugaucua augaucagca ggcaaagauu gugugcuguu
 781 ccaccuccca acuucgagau gccagucucc aucccagugu ccagccacaa caguuuggug
 841 uacagcaacc cugucagcuc acugggaaac cccaaccuau ugccacuggc ucacccuucu
 901 cugcagagga auaguauguc uccuggugua acacaucgac cuccaagugc agguaacaca
 961 gguggucuga ugggguggaga ccucacgucu ggugcaggca ccagugcagg gaacggguau
1021 ggcaaucccc gaaacucacc aggucugcug gucucaccug guaacuugaa caagaauaug
1081 caagcaaaau cuccuccccc aaugaauuua ggaaugaaua accguaaacc agaucuccga
1141 guucuuauuc caccaggcag caagaauacg augccaucag ugucugagga ugucgaccug
1201 cuuuugaauc aaaggauaaa uaacucccag ucggcucagu cauuggcuac cccagugguu
```

1261 uccguagcaa cuccuacuuu accaggacaa ggaaugggag gauauccauc agccauuuca

1321 acaacauaug guaccgagua cucucugagu agugcagacc ugucaucucu gucuggguuu

1381 aacaccgcca gcgcucuuca ccuugguuca guaacuggcu ggcaacagca acaccuacau

1441 aacaugccac caucugcccu cagucaguug ggagcuugca cuagcacuca uuuaucucag

1501 aguucaaauc ucucccugcc uucuacucaa agccucaaca ucaagucaga accuguuucu

1561 ccuccuagag accguaccac caccccuucg agauacccac aacacacgcg ccacgaggcg

1621 gggagaucuc cuguugacag cuugagcagc uguagcaguu cguacgacgg gagcgaccga

1681 gaggaucacc ggaacgaauu ccacuccccc auuggacuca ccagaccuuc gccggacgaa

1741 agggaaaguc ccucagucaa gcgcaugcga cuuucugaag gaugggcaac auga 3'UTR

Human *NKX2-5* mRNA: NM_004387.4

uORF sequence: 45-107 (63-nt)

mORF sequence: 124-1098 (975-nt)

uORF-ASO target sequence

mORF-ASO target sequence

1 acauccagag cuggccgacg ggugcgcggg cgggcggcgg caccaugcag ggaagcugcc

61 aggggccgug ggcagcgccg cuuucugccg cccaccuggc gcugugagac uggcgcugcc

121 accauguucc ccagcccugc ucucacgccc acgcccuucu cagucaaaga cauccuaaac

181 cuggaacagc agcagcgcag ccuggcugcc gccggagagc ucucugcccg ccuggaggcg

241 acccuggcgc ccuccuccug caugcuggcc gccuucaagc cagaggccua cgcugggccc

301 gaggcggcug cgccgggccu cccagagcug cgcgcagagc ugggccgcgc gccuucaccg

361 gccaagugug cgucugccuu ucccgccgcc cccgccuucu auccacgugc cuacagcgac

421 cccgacccag ccaaggaccc uagagccgaa aagaaagagc ugugcgcgcu gcagaaggcg

481 guggagcugg agaagacaga ggcggacaac gcggagcggc cccgggcgcg acggcggagg

541 aagccgcgcg ugcucuucuc gcaggcgcag gucuaugagc uggagcggcg cuucaagcag

601 cagcgguacc ugucggcccc cgaacgcgac cagcuggcca gcgugcugaa acucacgucc

661 acgcagguca agaucugguu ccagaaccgg cgcuacaagu gcaagcggca gcggcaggac

721 cagacucugg agcugguggg gcugccccg ccgccgccgc cgccugcccg caggaucgcg

781 gugccagugc uggugcgcga uggcaagcca ugccuagggg acucggcgcc cuacgcgccu

841 gccuacggcg ugggccucaa ucccuacggu uauaacgccu accccgccua uccggguuac

901 ggcggcgcgg ccugcagccc uggcuacagc ugcacugccg cuuaccccgc cgggccuucc

961 ccagcgcagc cggccacugc cgccgccaac aacaacuucg ugaacuucgg cgucggggac

1021 uugaaugcgg uucagagccc cgggauuccg cagagcaacu cgggaguguc cacgcugcau

1081 gguauccgag ccugguag 3'UTR

Human *eIF4G2* mRNA (isoform 1): NM_001418.4

uORF sequence: 23-73 (51-nt)

mORF sequence: 309-3032 (2724-nt)

uORF-ASO target sequence

mORF-ASO target sequence

1 gccagcagug agucggagcu cuauggaggu ggcagcgggu accgaguggc ggcugcagca

61 gcgacucccuc ugagcugagu uugaggccgu ccccgacucc uuccuccccc uucccucccc

121 cuuuuuuuug uuuuccguuc cccuuucccc ucccuucccu aucccccgacg accggauccu

181 gaggaggcag cugcgguggc agcugcugag uucucgguga agguauuuca uuucuccugu

241 ccccuccccu ccccaccccca ucuauuaaua uuauucuuuu gaagauucuu cguugucaag

301 ccgccaaagu ggagagugcg auugcagaag gggugcuuc ucguuucagu gcuucuucgg

361 gcggaggagg aaguaggggu gcaccucagc acuaucccaa gacugcuggc aacagcgagu

421 uccuggggaa aaccccaggg caaaacgcuc agaaauggau uccugcacga agcacuagac

481 gagaugacaa cuccgcagca aacaacuccg caaacgaaaa agaacgacau gaugcaaucu

541 ucaggaaagu aagaggcaua cuaaauaagc uuacuccuga aaaguuugac aagcuaugcc

601 uugagcuccu caaugugggu guagagucua aacucauccu uaaagggguc auacugcuga

661 uuguggacaa agcccuagaa gagccaaagu auagcucacu guaugcucag cuaugucugc

721 gauuggcaga agaugcacca aacuuugaug gcccagcagc agagggucaa ccaggacaga

781 agcaaagcac cacauucaga cgccuccuaa uuuccaaauu acaagaugaa uuugaaaacc

841 gaacuagaaa uguugauguc uaugauaagc gugaaaaucc ccuccucccc gaggaggagg

901 aacagagagc cauugcuaag aucaagaugu ugggaaacau caaauucauu ggagagcuug

961 gcaagcuuga ucuuauucac gaaucuaucc uucauaagug caucaaaaca cuuuuggaaa

1021 agaagaagag aguccaacuc aaagauaugg gagaggauuu ggagugccuc ugucagauaa

1081 ugaggacagu gggaccuaga uuagaccaug aacgagccaa guccuuaaug gaucaguacu

1141 uugcccgaau gugcuccuug auguuaagua aggaauugcc agcaaggauu cguuuccugc

1201 ugcaggauac cguagaguug cgagaacacc auuggguucc ucgcaaggcu uuucuugaca

1261 auggaccaaa gacgaucaau caaauucguc aagaugcagu aaaagaucua ggggguuuua

1321 uuccugcucc uauggcucaa gggaugagaa gugacuucuu ucuggaggga ccguucaugc

1381 cacccaggau gaaaauggau agggacccac uuggaggacu ugcugauaug uuuggacaaa

1441 ugccagguag cggaauuggu acugguccag gaguuaucca ggauagauuu ucacccacca

1501 ugggacguca ucguucaaau caacucuuca auggccaugg gggacacauc augccuccca

1561 cacaaucgca guuuggagag augggaggca aguuuaugaa aagccagggg cuaagccagc

1621 ucuaccauaa ccagagucag ggacucuuau cccagcugca aggacagucg aaggauaugc

1681 caccucgguu uucuaagaaa ggacagcuua augcagauga gauuagccug aggccugcuc

1741 agucguuccu aaugaauaaa aaucaagugc caaagcuuca gccccagaua acuaugauuc

1801 cuccuagugc acaaccacca cgcacucaaa caccaccucu gggacagaca ccucagcuug

1861 gucucaaaac uaauccaccg cuuauccagg aaaagccugc caagaccagc aaaaagccac

1921 caccgucaaa ggaagaacuc cuuaaacuaa cugaaacugu ugugacugaa uaucuaaaua

1981 guggaaaugc aaaugaggcu gucaauggug uaagagaaau gagggcuccu aaacacuuuc

2041 uuccugagau guuaagcaaa guaaucaucc ugucacuaga uagaagcgau gaagauaaag

2101 aaaaagcaag uucuuugauc aguuuacuca aacaggaagg gauagccaca agugacaacu

2161 ucaugcaggc uuuccugaau guauuggacc agugucccaa acuggagguu gacaucccuu

2221 uggugaaauc cuauuuagca caguuugcag cucgugccau cauuucagag cuggugagca

2281 uuucagaacu agcucaacca cuagaaagug gcacccauuu uccucucuuc cuacuuuguc

2341 uucagcaguu agcuaaauua caagaucgag aaugguuaac agaacuuuuu caacaaagca

2401 aggucaauau gcagaaaaug cucccagaaa uugaucagaa uaaggaccgc auguuggaga

2461 uuuuggaagg aaagggacug aguuucuuau ucccacuccu caaauuggag aaggaacugu

2521 ugaagcaaau aaaguuggau ccaucccuc aaaccauaua uaaauggauu aaagauaaca

2581 ucucucccaa acuucaugua gauaaaggau uugugaacau cuuaaugacu agcuucuuac

2641 aguacauuuc uagugaagua aacccccca gcgaugaaac agauucaucc ucugcuccuu

2701 ccaaagaaca guuagagcag gaaaaacaac uacuacuauc uuucaagcca guaaugcaga

2761 aauuucuuca ugaucacguu gaucuacaag ucagugcccu guaugcucuc caggugcacu

2821 gcuauaacag caacuuccca aaaggcaugu uacuucgcuu uuuugugcac uucuaugaca

2881 uggaaauuau ugaagaagaa gcuuucuugg cuuggaaaga agauauaacc caagaguuuc

2941 cgggaaaagg caaggcuuug uuccagguga aucaguggcu aaccugguua gaaacugcug

3001 aagaagaaga aucagaggaa gaagcugacu aa 3’UTR
