# Supplementary material for: Secondary structures that regulate mRNA translation provide insights for ASO-mediated modulation of cardiac hypertrophy
Source: Nat Commun. 2023 Oct 3;14:6166. doi: 10.1038/s41467-023-41799-1 (PMC10547706; doi:10.1038/s41467-023-41799-1)
Supplement: Supplementary file 12 — Supplementary Data 9 [file 41467_2023_41799_MOESM12_ESM.pdf]

Code for SHAPE normalization:

```
def NormSHAPE (filename, outputname, sequencesize):
#########################################################
#########################################################
######################    #step 1    #loading   packages
and file input    import numpy as np    import
csv    raw_data_file = open(filename,'r')    raw_data_line =
raw_data_file.readlines()##################################
#########################################################
##############################################    #step 2
will turn the tsv file into a list and sort it    list_of_reactivities =
[]    for i in raw_data_line:        line_cleaned = i.replace("\n",
"")        line_in_file =
line_cleaned.split("\t")        line_in_file_numerical = [float(i) for i
in line_in_file] #convert each list to numerical values so I can sort
them
later        #print(line_in_file_numerical)        list_of_reactivitie
s.append(line_in_file_numerical)    sorted_reactivities =
sorted(list_of_reactivities, key=lambda
x:x[1])#################################################
#########################################################
###############################    #I decided to split the list
of lists (i.e. list of: [nucleotide number, raw value]) into 2 separate
lists    sorted_reactivities_zeroth_element = []    for i in
sorted_reactivities:        sorted_reactivities_zeroth_element.app
end(i[0])    sorted_reactivities_first_element = []    for i in
```

```
sorted_reactivities:        sorted_reactivities_first_element.appen
d(i[1])  #the aim here is to have 2 lists that map to each other
##########################################################
##########################################################
#######################    #step 3 calculate the quartiles and
interquartile distance    #calculate the quartiles    Q1_react=
np.quantile(sorted_reactivities_first_element, 0.25, axis =
None)    Q3_react =
np.quantile(sorted_reactivities_first_element, 0.75, axis =
None)    Interquart_dist = Q3_react -
Q1_react    reactivity_list_size =
len(sorted_reactivities_first_element)    outliers_removed =
sorted_reactivities_first_element    outliers_removed_indices =
sorted_reactivities_zeroth_element    outliers_removed_indices
_int = [str(int(i)) for i in outliers_removed_indices] #fixed because
RNA structure expects
integers#################################################
##########################################################
################################    #step 4 removing
outliers    percentage_int = int()  #if the sequence us > or = to
100 we can remove up to 10% otherwise we remove maximum
5%    if sequencesize  >= 100:        percentage_int =
0.90    else:        percentage_int = 0.95    while
(len(outliers_removed)/reactivity_list_size) >
percentage_int:        #print(len(outliers_removed)/reactivity_list
_size)        #print(Q3_react + (1.5 * Interquart_dist))        if
sorted_reactivities_first_element[len(outliers_removed) - 1] >
```

(Q3_react + (1.5 *
Interquart_dist)): outliers_removed.pop() outlie
rs_removed_indices_int.pop() #remember we split the list of lists
into two
lists else: break ########################
##
##
#step 5 calculating RMax and calculating the normalized
values top_ten_percent_list = [] top_ten_percent_react =
np.quantile(outliers_removed, 0.9, axis = None) for i in
outliers_removed: if i >=
top_ten_percent_react: top_ten_percent_list.append(i)
R_Max = sum(top_ten_percent_list) /
len(top_ten_percent_list) #print(R_Max) normalized_list
= [] for j in outliers_removed: normalized_list.append(j
/ R_Max) list_of_rows = []
##
##
###################### #output step for i in range(0,
len(normalized_list)): next_row =
[outliers_removed_indices_int[i],
normalized_list[i]] list_of_rows.append(next_row) #
writing to csv file with open(outputname, 'w') as
tsvfile: # creating a csv writer object tsvwriter =
csv.writer(tsvfile, delimiter='\t') # writing the data
rows tsvwriter.writerows(list_of_rows) ###a = "INSERT
PATH HERE" ###b = "FILEOUTPUT.txt" #RUN FUNCTION

BELOW AS SHOWN###NormSHAPE(a, b, 200)
