# Supplementary material for: Secondary structures that regulate mRNA translation provide insights for ASO-mediated modulation of cardiac hypertrophy
Source: Nat Commun. 2023 Oct 3;14:6166. doi: 10.1038/s41467-023-41799-1 (PMC10547706; doi:10.1038/s41467-023-41799-1)
Supplement: Supplementary file 13 — Reporting Summary [file 41467_2023_41799_MOESM13_ESM.pdf]

nature portfolio

Corresponding author(s): Peng Yao

Last updated by author(s): Aug 14, 2023

Reporting Summary

Nature Portfolio wishes to improve the reproducibility of the work that we publish. This form provides structure for consistency and transparency in reporting. For further information on Nature Portfolio policies, see our Editorial Policies and the Editorial Policy Checklist.

Statistics

For all statistical analyses, confirm that the following items are present in the figure legend, table legend, main text, or Methods section.

n/a	Confirmed	
☐	☒	The exact sample size (*n*) for each experimental group/condition, given as a discrete number and unit of measurement
☐	☒	A statement on whether measurements were taken from distinct samples or whether the same sample was measured repeatedly
☐	☒	The statistical test(s) used AND whether they are one- or two-sided<br>*Only common tests should be described solely by name; describe more complex techniques in the Methods section.*
☒	☐	A description of all covariates tested
☒	☐	A description of any assumptions or corrections, such as tests of normality and adjustment for multiple comparisons
☐	☒	A full description of the statistical parameters including central tendency (e.g. means) or other basic estimates (e.g. regression coefficient) AND variation (e.g. standard deviation) or associated estimates of uncertainty (e.g. confidence intervals)
☐	☒	For null hypothesis testing, the test statistic (e.g. *F*, *t*, *r*) with confidence intervals, effect sizes, degrees of freedom and *P* value noted<br>*Give P values as exact values whenever suitable.*
☒	☐	For Bayesian analysis, information on the choice of priors and Markov chain Monte Carlo settings
☒	☐	For hierarchical and complex designs, identification of the appropriate level for tests and full reporting of outcomes
☐	☒	Estimates of effect sizes (e.g. Cohen's *d*, Pearson's *r*), indicating how they were calculated

Our web collection on statistics for biologists contains articles on many of the points above.

Software and code

Policy information about availability of computer code

Data collection	SAFA (Measuring band intensity on RNA SHAPE gel); The polysome software: PeakChart from Brandel company; confocal: FV10-ASW4.0 from Olympus; qPCR: CFX Maestro software from Bio-rad; gene sequence information: Ensembl genome browser and National Center for Biotechnology Information; echocardiography data collection: Vevo LAB (Fuji VisualSonics, https://www.visualsonics.com/product/software/vevo-lab)
Data analysis	EXCEL (Data arrangement and manipulation); Graph Pad Prism (statistical analysis and data plotting); Custom Code (Python; analyzing RNA SHAPE values); RNAfold web server (RNA secondary structure prediction and implementation of SHAPE values)<br>Code for SHAPE normalization is available in accompanying Supplementary Information file.

For manuscripts utilizing custom algorithms or software that are central to the research but not yet described in published literature, software must be made available to editors and reviewers. We strongly encourage code deposition in a community repository (e.g. GitHub). See the Nature Portfolio guidelines for submitting code & software for further information.

Data

Policy information about availability of data

All manuscripts must include a data availability statement. This statement should provide the following information, where applicable:

- Accession codes, unique identifiers, or web links for publicly available datasets
- A description of any restrictions on data availability
- For clinical datasets or third party data, please ensure that the statement adheres to our policy

Data availability
Further information and requests for resources and reagents should be directed to, and will be fulfilled by, the Lead Contact, Peng Yao (peng_yao@urmc.rochester.edu). All stable reagents generated in this study are available from the Lead Contact without restriction, or with a Materials Transfer Agreement. All Complete gel and blot images for main and supplementary figures are available in accompanying Supplementary Information file. Source data are provided with this paper. All graph data used in this study are available in the accompanying Source Data file. All nucleotide sequences used in the manuscript are available in the Supplemental Information or NCBI Nucleotide database. Sequences of the human mRNA 5' UTRs in Fig. 1a, b were retrieved from GRCh38.p13 (https://ftp.ebi.ac.uk/pub/databases/gencode/Gencode_human/release_40/gencode.v40.transcripts.fa.gz).

Research involving human participants, their data, or biological material

Policy information about studies with human participants or human data. See also policy information about sex, gender (identity/presentation), and sexual orientation and race, ethnicity and racism.

Reporting on sex and gender	NA
Reporting on race, ethnicity, or other socially relevant groupings	NA
Population characteristics	NA
Recruitment	NA
Ethics oversight	NA

Note that full information on the approval of the study protocol must also be provided in the manuscript.

Field-specific reporting

Please select the one below that is the best fit for your research. If you are not sure, read the appropriate sections before making your selection.

☒ Life sciences ☐ Behavioural & social sciences ☐ Ecological, evolutionary & environmental sciences

For a reference copy of the document with all sections, see nature.com/documents/nr-reporting-summary-flat.pdf

Life sciences study design

All studies must disclose on these points even when the disclosure is negative.

Sample size	Biochemical and cellular assay sample sizes were routinely performed in duplicates or triplicates. In animal studies, a pilot study is conducted and based on the results the minimum sample size is determined. Sample size was determined to be adequate to lead to meaningful conclusions based on the magnitude and consistency of measurable differences. Our number of data points have proven to be sufficient evident by the statistical significance. The sample size (n) of each experiment is provided in the corresponding figure legends in both main manuscript (main figures) and supplementary figures.
Data exclusions	No data was excluded.
Replication	All in vitro experiments were repeated successfully at least 2-3 times. In vivo experiments based on genetic or disease mouse models were done in biological replicates and sample size calculations and statistical analysis were performed (described for each experiment the figure legends or methods).
Randomization	Wild-type C57BL/6J mice with the same genetic background followed by random selection were used for animal studies. Randomization is not relevant to our studies as there are no known variables or biases in the wild-type C57BL/6J mice with the same genetic background. Mice used and analyzed were age- and sex-matched litter mates.
Blinding	The person injecting the mice (volunteer) is separate from the person harvesting the samples (investigator). The volunteer removed cage labels and replaced them with numbers. The investigator then harvested the mouse samples in a blinded manner. Echocardiography measurement and analysis were independently done by vivarium surgeons from the Microsurgical Core Facility and they are blinded to genotypes. The same double blind principle also applies to histological analysis.

Reporting for specific materials, systems and methods

We require information from authors about some types of materials, experimental systems and methods used in many studies. Here, indicate whether each material, system or method listed is relevant to your study. If you are not sure if a list item applies to your research, read the appropriate section before selecting a response.

Materials & experimental systems

n/a	Involved in the study
☐	☒ Antibodies
☐	☒ Eukaryotic cell lines
☒	☐ Palaeontology and archaeology
☐	☒ Animals and other organisms
☒	☐ Clinical data
☒	☐ Dual use research of concern
☒	☐ Plants

Methods

n/a	Involved in the study
☒	☐ ChIP-seq
☒	☐ Flow cytometry
☒	☐ MRI-based neuroimaging

Antibodies

Antibodies used	Rabbit anti-GATA4 (19530-1-AP; ProteinTech), Rabbit anti-NKX2-5 (13921-1-AP; ProteinTech), Mouse anti-a-Actinin (A7811; Sigma Aldrich), Mouse anti-MEF2C (365862; Santa Cruz), Mouse anti-β-actin (MA5-11869; Invitrogen), Mouse anti-DAP5 (135999; Santa Cruz), Sheep anti-mouse IgG (NXA931V; Roche), Donkey anti-rabbit IgG (NA9340V; Roche), Goat anti-rabbit-AF-488 (A-11001; Invitrogen), Rabbit anti-DDX3X antibody (HPA005631-100UL; Sigma Aldrich), Normal rabbit IgG (2729S; Cell Signaling)
Validation	All antibodies are commercial. We collected information from public website (https://www.labome.com/index.html) to choose the antibody which has been used multiple times from literature. We also carefully examined the data sheet from the vendor website. We then validated the antibody using our own cell culture and siRNA knockdown.

Eukaryotic cell lines

Policy information about cell lines and Sex and Gender in Research

Cell line source(s)	HEK293T cells (ATCC #CRL-3216), H7 hESCs (WiCell #WA07), and AC16 (Sigma #SCC109)
Authentication	AC16 was purchased from Millipore-Sigma as a fresh cell line. HEK293T cells were purchased from ATCC. H7 hESCs were obtained from our co-author Dr. Chris Proschel's lab. None were authenticated in our lab.
Mycoplasma contamination	Mycoplasma testing is carried out once a year. Contaminated cell-lines are not used.
Commonly misidentified lines (See ICLAC register)	None were used.

Animals and other research organisms

Policy information about studies involving animals; ARRIVE guidelines recommended for reporting animal research, and Sex and Gender in Research

Laboratory animals	This study used wild-type (WT) C57BL/6J mice (Jackson Laboratories). Mice were maintained on a 12-hour light/dark cycle and fed with a normal chow diet and water at 22°C with 40-60% humidity in a vivarium facility. C57BL/6J wild type mice are between 10-12 weeks old. Experimental mice are siblings generated from intercrosses of wild type mice. Both male and female mice with matched age were used.
Wild animals	None were used.
Reporting on sex	Both sexes were equally represented in our animal studies. We did not test for sex-specific effects because it is outside the scope of the current work.
Field-collected samples	None were used.
Ethics oversight	C57BL/6J mice of the same age (10-12 weeks) and gender (male and female) from littermates or sibling mating were used for experiments with WT mice. All animal procedures were performed in accordance with the National Institutes of Health (NIH) and the University of Rochester Institutional guidelines. The University of Rochester Medical Center Animal Care and Use of Committee approved all experimental animal procedures.

Note that full information on the approval of the study protocol must also be provided in the manuscript.
